# Medicaid Education and Eligibility Planning for Caregivers: Website Usability and Validation Study

**DOI:** 10.2196/77441

**Published:** 2025-08-27

**Authors:** Marguerite DeLiema, Siyu Gao, Justine Scattarelli, Kelly Moeller, Olu Olofinboba

**Affiliations:** 1 School of Social Work, University of Minnesota, 1404 Gortner Ave., Saint Paul, MN, 55455, United States, 1 6126258798; 2 Department of Educational Psychology, University of Minnesota, Minneapolis, MN, United States; 3 School of Public Health, University of Minnesota, Minneapolis, MN, United States; 4 Moai Technologies (United States), Minneapolis, MN, United States

**Keywords:** long-term services and supports, long-term care, Medicaid waiver, user-centered design, cost of care, financial education, Medicaid planning, caregiver education, web application

## Abstract

**Background:**

Most older Americans have not saved enough to cover long-term care costs. Medicaid—a public health care program for low-income individuals—can help Americans with qualifying care needs pay for assistance in a nursing home or for services in the home. Determining financial eligibility for Medicaid is complicated, and the application process is often managed by family caregivers with limited knowledge of Medicaid programs.

**Objective:**

A one-stop digital solution is needed to help family caregivers plan for the cost of long-term care services and learn about getting help paying for services through Medicaid. We aimed to develop a web application that (1) educates informal caregivers about Medicaid programs and eligibility criteria, (2) informs them about the cost of home and institutional care in their local area with and without Medicaid coverage, and (3) uses a custom algorithm to provide personalized financial eligibility information based on the care recipient’s income, assets, and monthly spending.

**Methods:**

We first interviewed aging services providers and informal family caregivers, then developed a web application that was refined based on user experience interviews with English- and Spanish-speaking caregivers. In the final validation phase, asynchronous usability sessions were recorded with 109 informal caregivers who completed a series of tasks. Participants viewed and rated animated Medicaid “explainer” videos, input financial information to enable the custom algorithm to determine the care recipient’s eligibility for Medicaid, adjusted settings on a care cost calculator to estimate the regional cost of home and institutional care services, and completed a Medicaid knowledge quiz before and after using the website.

**Results:**

After engaging with the website and watching the videos, scores on a Medicaid knowledge quiz increased by 61.2% (2-tailed *t*_92_=12.9, *P*<.001). Participants found it easy to enter the care recipient’s financial information to determine Medicaid eligibility (out of 7; mean 5.9, SD 1.3) and perceived the care cost calculator as very helpful (out of 7; mean 6.3, SD 1.2). The website received a very high System Usability Scale rating of 88.3 out of 100 (SD 13.1). Caregivers verbalized wanting more education on complex financial concepts that impact Medicaid eligibility and asset preservation.

**Conclusions:**

A comprehensive Medicaid planning website can significantly improve caregivers’ knowledge of Medicaid and provide them with a personalized roadmap for accessing care services. The custom algorithm powering the Medicaid eligibility determination could be further refined to account for state-based exceptions. This application may reduce caregiver burden and help support the long-term care planning process.

## Introduction

### Background

Approximately 70% of Americans need long-term services and supports (LTSS) as they age, and 48% use some form of paid care such as skilled nursing, assisted living, home health, and adult day services [1]. The likelihood of needing LTSS and the overall cost are much greater for those living with Alzheimer disease and related dementias (ADRD) [2-4], which affects approximately 1 in 10 adults aged 65 years and older [5]. Based on a 2024 survey of long-term care providers in the United States, the median cost of care in a skilled nursing facility exceeds US $110,000 per year, whereas assisted living facilities cost approximately US $70,000, and a 44-hour per week home health aide is US $78,000 [6].

Most older adults have not saved enough to cover these high costs, and only 11% have purchased private long-term care insurance [7,8]. In a national survey, Hamel and Montero [7] found that only 23% of adults aged 50-64 years reported that they set aside money to pay for future expenses, and 83% stated that it would be impossible or very difficult to pay for a home health aide or a 1-year stay in a nursing home. As a result, most Americans rely on informal (unpaid) family and friend caregivers until their support needs reach a point when formal LTSS is needed [9].

### Medicaid as a Primary Payer of LTSS

Due to the high cost of formal LTSS, many Americans turn to Medicaid, a joint state and federal health insurance program for low-income individuals [10]. Medicaid is the nation’s primary payer of LTSS; approximately one-third of total program costs go toward LTSS [11]. In 2020, 62% of nursing home residents were covered by Medicaid [12]. Traditionally, Medicaid reimburses institutional care provided in skilled nursing facilities, but following the 1981 Omnibus Budget Reconciliation Act, Medicaid waivers allowed states to expand coverage to home and community-based services (HCBS), such as home health and personal care aides, case management, caregiver respite, and adult day services. In 2022, 87% of Medicaid LTSS users received HCBS [13].

Unfortunately, many older adults struggle to access Medicaid benefits. For one, Americans hold misconceptions about LTSS insurance coverage; 41% falsely believe that Medicare, the health insurance program for adults aged 65 years and older, covers long-term stays in a nursing home [7]. Older adults may underestimate the cost of residential and home care services or believe they can rely on family members for assistance [14,15]. These assumptions can lead eligible older adults to delay Medicaid enrollment.

Navigating formal LTSS options is a high-stakes, multistep process that often includes touring facilities, interviewing home health aides, calculating costs, and determining whether the care recipient is eligible for public benefits. Because of physical and cognitive impairments, these activities often fall on informal family caregivers who are encountering public benefit programs for the first time. The decision to apply for Medicaid may be triggered by a health crisis, compounding the emotional and cognitive burden of caregiving [15]. When helping the older adult apply for Medicaid, informal caregivers encounter unfamiliar terms like “spousal resource allowance” (the amount of household assets a Medicaid applicant’s spouse is allowed to keep) and “asset spend down” (the process of drawing down one’s savings and investments to meet eligibility limits).

State-to-state variability in Medicaid eligibility criteria is an additional challenge to understanding who qualifies. For example, in states such as Iowa, Maine, and New Mexico, an individual applying for Medicaid must have less than US $2000 in assets; in New York, the asset limit is US $32,396. California eliminated the asset limit in 2024 [16]. Some states offer exceptions for certain types of assets, and many provide alternative pathways to qualify when assets or income exceed state limits. This lack of standardization in Medicaid policy may cause confusion, especially among informal caregivers who reside in a different state than the care recipient they are helping to enroll.

The demands of the Medicaid application process itself may serve as a significant barrier to enrollment. Moynihan et al [17] argue that “by constructing complex, confusing, and time-consuming application procedures, the state can effectively thwart an individual from accessing benefits, even if eligible by law.” Several factors must come together for the application to be processed successfully. First, the care recipient must undergo a functional needs assessment that is scheduled with and administered by the proper local authority. They must submit recent financial and insurance documentation to the state or county. This information can be difficult to obtain by informal caregivers who may lack the legal authority to access accounts. Also, most states have a “look-back” period of 60 months (5 y) that penalizes applicants who gifted or transferred assets to family, friends, or charities before applying, further delaying the eligibility [16].

Researchers have called for digital solutions (“digital nudging”) and more automation to reduce information overload and ease the cognitive burden of applying for government programs [18,19]. Digital nudging involves creating user-friendly interface designs that guide behavior in digital choice environments [20]. Effective digital nudges can help caregivers make better choices by simplifying their choice sets; for example, restricting the list of formal LTSS services to those that accept Medicaid clients or only presenting information on spousal resource allowances if the care recipient is married.

Systematic reviews suggest that informal family caregivers are motivated to use technology to help manage caregiving challenges [21-24]. Web and mobile applications have been developed to improve caregiver well-being and resilience [25], motivate physical activity [26], coordinate care [27], and engage with peer support [28], but to date, there is no one-stop solution designed for older adults and their caregivers to get personalized information on offsetting the high cost of institutional LTSS or HCBS by enrolling in Medicaid.

### Study Objective

To address this resource gap, the goal of this web application is to (1) educate informal caregivers about Medicaid programs and eligibility criteria, (2) improve caregivers’ understanding of the local costs of LTSS with and without Medicaid coverage, and (3) provide personalized eligibility timelines based on the applicant’s state of residence, income, assets, and monthly spending. In this study, we present the findings from the asynchronous mixed methods website validation test with 109 current and former older adult caregivers. We assessed their Medicaid program and eligibility knowledge before and after interacting with the website in a pretest-posttest design.

## Methods

### Development of the Web Application

**Figure 1.**
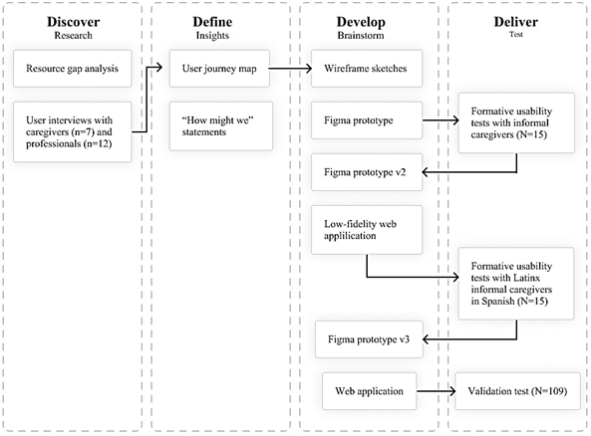
Research and web application development steps.

Website development (Figure 1) followed the Double Diamond design-thinking process, which is a user-centered approach to problem solving comprising 4 iterative phases: discover, define, develop, and deliver [29]. As part of the “discover” stage, the research team interviewed informal caregivers and aging services providers (eg, certified Medicaid planners, county financial workers, geriatric care managers, and social workers) to better understand the challenges of navigating LTSS options, costs, and the process of applying for Medicaid and Medicaid waivers [15]. Involving informal caregivers and direct service providers in the early stages of design is critical to ensuring the end product addresses their stated needs [22].

In the “define” stage, a larger team of researchers, developers, and designers participated in a synchronous problem definition session and created a user journey map and user personas (Multimedia Appendix 1) to visualize the process of accessing and financing LTSS, as uncovered by our initial research. The team wrote “how might we” statements to further define the problem and begin to conceptualize how a digital tool could support caregivers (eg, “How might we inform caregivers that home care services are an option?”).

Alternating “design” and “develop” activities followed. First, team members sketched wireframes of possible solutions and voted on an initial concept. Designers then built a prototype using Figma, a collaborative design software. Researchers tested this first iteration with 15 informal caregivers for older adults with ADRD. The 90-minute formative usability tests were conducted via Zoom (Zoom Communications). Based on these findings, a second iteration of the design was created to include larger information displays, a sidebar menu, and cursor hover capabilities. This version was developed into a dual-language (English and Spanish) web application and was tested by 15 Spanish-speaking informal caregivers. These formative usability tests were moderated by Spanish-speaking interviewers, also using Zoom.

### Overview of Web Application

**Figure 2.**
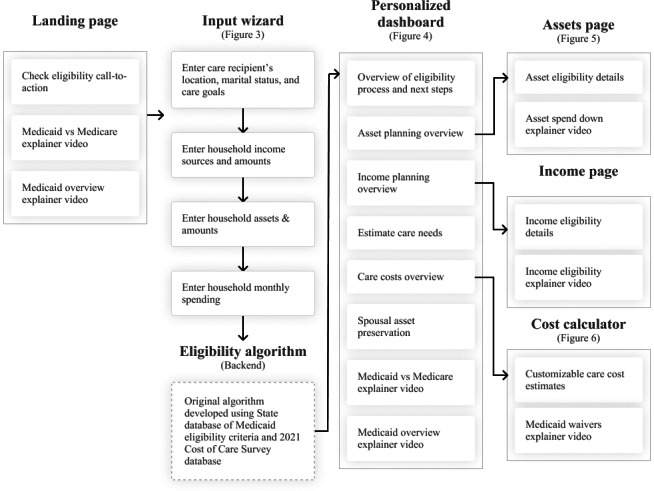
Visualization of the web application—navigation, key pages, and functions.

**Figure 3.**
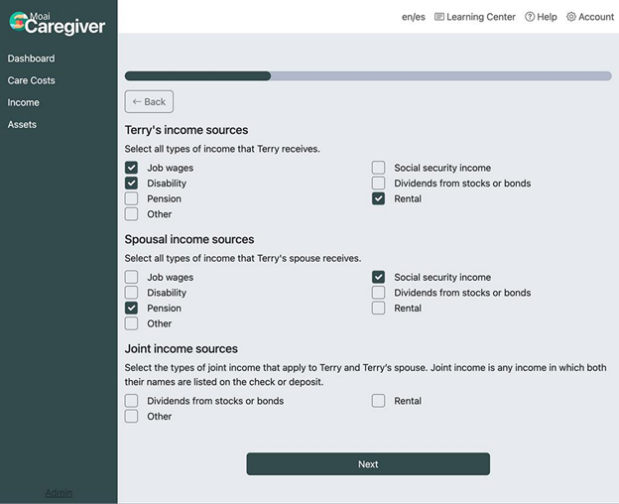
The “select income sources” step of the input wizard.

Participant feedback from both user experience test phases was incorporated into a final design. A high-level representation of the final flow of the web application is presented in Figure 2. The landing page welcomes users with a large image and the message: “Get help paying for long-term care. If you don’t qualify now, you might in the future. We’ll help you get there.” From the landing page, users can click the “Check Eligibility” button that takes them through an input wizard where they enter information about the care recipient (ie, applicant): name, location, marital status, and care goals (independent, assisted living, and nursing home). The user is then asked to select the types and enter values (in US $) of the care recipient’s income sources (Figure 3), assets, and monthly expenses. The input wizard collects information about the applicant’s income and assets separately from their spouse’s (if applicable) because nearly all states exempt spousal income sources from the applicant’s eligibility determination. In some states, such as Delaware and Wisconsin, the spouse’s retirement accounts (Individual Retirement Accounts and 401k) are also exempt.

User data collected during the input phase is then passed through an original Medicaid eligibility algorithm that uses a database of state-specific Medicaid eligibility criteria. This database includes information about each state’s income and asset limits, personal needs allowances, home equity amounts, options to qualify when over limits, Medicaid “look-back” periods, and community spouse asset allowances and monthly maintenance needs allowances. The database is also programmed with various state-specific exceptions and thresholds for certain asset types, such as retirement accounts and the equity interest limit of the applicant’s primary home (eg, US $730,000 in most states). The algorithm sums and compares the values entered by the user with the information in the state eligibility database to (1) determine if the applicant is currently eligible for Medicaid because they are below their state’s (a) income limit, and (b) asset limit. If assets are higher than the eligibility limit, the algorithm calculates the amount of the applicant’s asset spend-down obligation (current countable assets minus the state’s asset limit) and the spousal resource allowance, if applicable (either 50% of total household assets or 100% up to the maximum amount allowed by the state, typically US $157,920).

**Figure 4.**
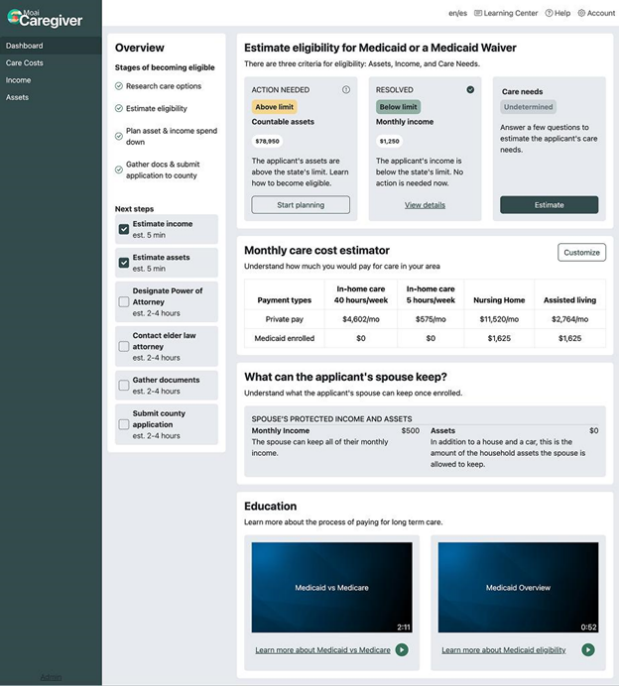
The dashboard of the web application.

Upon completing the input wizard, users arrive at the Dashboard page that presents a personalized Medicaid eligibility summary (Figure 4). The Dashboard shows whether the care recipient is above or below the state’s income and asset limit. It also provides a summary of average out-of-pocket home and institutional care costs with and without Medicaid enrollment and a checklist of next steps for applying, for example, “contact county Medicaid office” and “gather account statements.” If the care recipient is married, the Dashboard also provides information on the amount of monthly income and assets their community-residing spouse can keep as a resource allowance.

**Figure 5.**
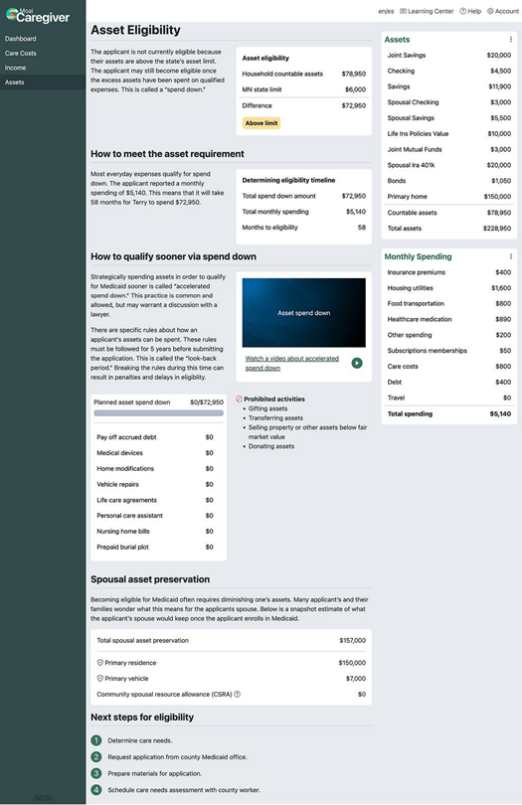
The “Asset Eligibility” page.

From the Dashboard, users can navigate to the “Assets” page (Figure 5), which displays the amount the care recipient must spend down to meet their state’s Medicaid asset limit, excluding exempt assets such as personal belongings and the spousal resource allowance (if applicable). Text with simple graphics informs users about qualified expenses (eg, care services, prepaid burial plots, and home renovations to improve accessibility) and prohibited expenses that violate Medicaid’s “look-back” period for gifts and unauthorized asset transfers.

**Figure 6.**
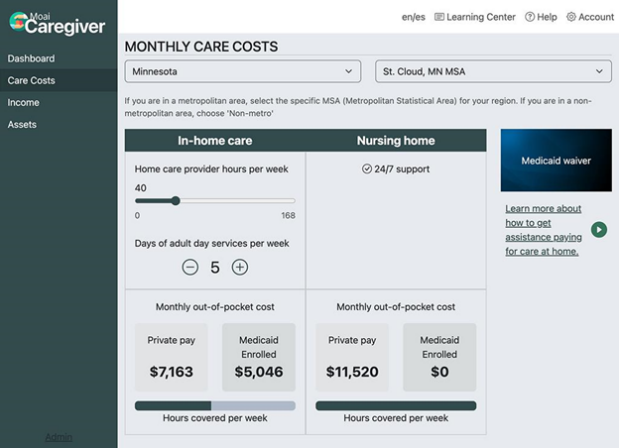
The “care cost calculator” of the web application.

Users can also navigate to the “Income” page, which provides personalized information about the state’s income eligibility limits, a summary of the care recipient’s monthly income sources, and how they can still qualify for Medicaid if their monthly income is above the state’s limit. The care cost calculator—accessible from the Dashboard, menu, or the landing page—displays the average monthly cost of different types of LTSS in the applicant’s local region (Figure 6). Out-of-pocket costs for private pay are compared to out-of-pocket costs for Medicaid-enrolled individuals. The calculator uses the Federal Long Term Care Insurance Program 2021 Cost of Care Survey database to estimate costs by Metropolitan Statistical Areas (MSAs) for (1) nursing home, private room; (2) home health aide (hourly); and (3) nonmedical adult day care (per day) [30]. For states where adult day care estimates were missing from the Federal Long Term Care Insurance Program database, and for homemaker services, we used an estimate for each state based on the November 2021 Genworth Cost of Care Survey [6]. Monthly out-of-pocket cost estimates are calculated by combining the Medicaid eligibility criteria with data on the cost of care in the region selected by the user. A slider bar allows users to adjust the hours of care provided by a home health aide and add or subtract the number of days of nonmedical adult day care. Average monthly costs of nursing home care (with and without Medicaid coverage) are presented next to the costs of HCBS.

Short, animated videos are contextualized throughout the website to educate users on basic Medicaid facts, including what services may be covered and information about eligibility and spousal resource allowances. Videos were generally less than 1.5 minutes long and covered the following topics: Medicaid versus Medicare, Medicaid eligibility overview, Medicaid waivers, asset spend down, and income eligibility.

After development, the research team led an asynchronous validation study of the final version of the web application with English-speaking informal caregivers of adults aged more than 60 years. This paper presents those results.

### Participants

Usability testing was performed using Userfeel (Userfeel Ltd), an online platform where participants record their screens and provide verbal feedback as they navigate websites and complete defined tasks. All Userfeel panel members have screen-capture and audio recording software installed on their computers and are familiar with the testing platform. Sessions were conducted remotely with no live facilitator.

Current and former caregivers of older adults were recruited from Userfeel’s participant panel (N=112). Only Userfeel panel members who passed the screening questionnaire were invited to participate. Recruitment criteria included (1) resident of the United States, (2) aged 18 years or older, (3) fluent in English, (4) using a desktop or laptop computer for the session, and (5) care (or cared) for an older family member or friend aged more than 60 years. Caregiving was defined as “regularly preparing meals, providing transportation, managing household tasks, monitoring medication, helping manage money, and assisting with personal care.”

### Procedure

Study procedures were reviewed and approved by the University of Minnesota Institutional Review Board. Participants who passed the screening questions and consented to the study were routed to the website. Task instructions and usability questions appeared in a small window in the upper right corner of their computer screens (Multimedia Appendix 2). Participants could minimize the task window but were not able to return to previous tasks or change their answers once they progressed. Participants were instructed to “think aloud” [31] as they navigated the website, providing verbal commentary that was recorded and transcribed by Userfeel. Participants could pause the recording and timer as desired.

Before exploring the landing page, participants answered 11 true or false and multiple-choice questions that assessed their current knowledge of Medicaid LTSS programs and eligibility criteria. These questions appeared in the task window. Participants were told not to refer to the website or to search the internet for answers. After the pretest, participants were presented with a series of tasks which included watching and rating the helpfulness of 4 of the 6 explainer videos, using the intake wizard to enter the care recipient’s income, assets, and monthly spending to determine their eligibility for Medicaid, and adjusting the care cost calculator settings to determine average HCBS and institutional care costs if the person was (1) Medicaid-enrolled or (2) a private pay user.

As they followed task instructions and progressed through the website, participants rated their agreement with various statements describing the website’s trustworthiness, credibility, attractiveness, the usefulness of specific features, and other characteristics. Responses ranged from 1=“strongly disagree” to 5=“strongly agree.” After answering the user satisfaction questions, participants were presented with a 10-item posttest on Medicaid LTSS programs and eligibility.

The posttest was followed by a brief demographic and caregiver questionnaire that asked participants about their relationship to the care recipient and whether the care recipient was enrolled in Medicaid or a Medicaid waiver program. A final question asked participants to verbalize any final overall impressions of the website. The recording ended after participants closed the final task window or after the timer reached 40 minutes, regardless of whether they finished the tasks.

Participants completed the System Usability Scale (SUS) immediately after the session ended. The SUS is a 10-item scale that asks participants the extent to which they agree or disagree with statements such as, “I thought the system was easy to use” and “I thought there was too much inconsistency in this system.” Half of the items are reverse scored, and total scores range from 1 to 100 [32].

### Analysis

Researchers reviewed all screen and audio recordings to evaluate session quality, export quotes into a spreadsheet, and document task completion rates (success or fail). Although 112 total sessions were recorded, 3 sessions were dropped from the final sample because they involved the same participant who used a virtual private network (VPN) to enter the study multiple times. Participating in the same usability test more than once is prohibited by Userfeel and the study protocol. Of the remaining 109 participants, 93 people were able to complete all of the Medicaid knowledge pretest and posttest questions, and 89 finished all tasks within the allotted 40 minutes.

Participants’ ratings of website features and task completion rates were analyzed descriptively. A paired-samples *t* test was used to determine changes in Medicaid knowledge after engaging with the website and watching the explainer videos. We also analyzed whether the change in knowledge significantly differed between those with and without previous Medicaid experience using an independent samples *t* test. Participants who ran out of time before finishing the Medicaid knowledge posttest did not have their scores included in the analysis. Verbal feedback, including direct quotes, was organized in a spreadsheet according to the website feature.

### Ethical Considerations

The protocol was reviewed and approved by the University of Minnesota Institutional Review Board (approval ID STUDY00013179). All participants were presented with the informed consent form during screening. They were required to read and agree to the study procedures before they could begin. No personally identifying information was collected, and all study records were stored securely and accessible only to the research team. All participants were compensated US $30, and the information they entered into the website was deleted from the database after the study period.

## Results

### Participant Characteristics

Participant sex and age are collected by Userfeel as part of their onboarding process. As illustrated in Table 1, out of 109 participants, 64 (58.7%) identified as female. The average age was 43.6 (SD 12.8) years. Other demographic and caregiving information, which was collected last, is missing from 17 participants (15.6%) who ran out of time before reaching these final questions. Furthermore, 56 participants (51.4%) reported their race as White, 21 (19.3%) as Black or African American, 4 (3.7%) as Asian, 10 (9.2%) as American Indian, multiple races, or “other,” and 2 participants (1.8%) preferred not to state. In addition, 12 participants (11%) were Hispanic or Latinx. Of the total, 14 participants (12.8%) reported an annual household income of less than US $25,000, 23 (21.1%) had household incomes between US $25,001 and US $50,000, 35 (32.1%) between US $50,001 and US $100,000, and 20 (18.4%) reported incomes greater than US $100,001.

**Table 1.** Participant characteristics (N=109).

Characteristic	Participants
Age (y), mean (SD)	43.6 (12.8)
Sex, n (%)	
Male	44 (40.4)
Female	64 (58.7)
Transgender	1 (0.9)
Race, n (%)	
White or Caucasian	56 (51.4)
Black or African American	21 (19.3)
Asian	4 (3.7)
American Indian or Alaska Native	1 (0.9)
Mixed race or other	9 (8.3)
Prefer not to answer	2 (1.8)
Missing	16 (14.7)
Ethnicity, n (%)	
Hispanic or Latinx	12 (11)
Non-Hispanic or Latinx	81 (74.3)
Missing	16 (14.7)
Income (US $), n (%)	
200,001-300,000	4 (3.7)
150,001-200,000	3 (2.8)
100,001-150,000	13 (11.9)
75,001-100,000	18 (16.5)
50,001-75,000	17 (15.6)
25,001-50,000	23 (21.1)
Under 25,000	14 (12.8)
Missing	17 (15.6)
Care recipient relationship, n (%)	
The care recipient is or was another relative	6 (5.5)
The care recipient is or was my aunt/uncle	3 (2.8)
The care recipient is or was my friend	5 (4.6)
The care recipient is or was my grandparent or grandparent-in-law	30 (27.5)
The care recipient is or was my spouse or partner	1 (0.9)
The care recipient is or was parent or parent-in-law	47 (43.1)
Missing	17 (15.6)
Length of providing care, n (%)	
Greater than 10 years	5 (4.6)
Between 6 and 10 years	10 (9.2)
Between 4 and 6 years	11 (10.1)
Between 2 and 4 years	21 (19.3)
Between 1 and 2 years	22 (20.2)
6 months to 1 year	17 (15.6)
Less than 6 months	6 (5.5)
Missing	17 (15.6)

In total, 47 participants (43.1%) reported that they care or cared for a parent or parent-in-law, 30 (27.5%) care or cared for a grandparent or grandparent-in-law, 6 (5.5%) care or cared for another relative, and 5 (4.6%) care or cared for a friend. Only one person cares or cared for a spouse. Furthermore, 28 participants responded that the person they care or cared for was enrolled in Medicaid or received a Medicaid waiver for HCBS, 44 (41.3%) were not Medicaid-enrolled, 12 (11%) were unsure, and 9 (14.7%) were missing. Nearly half (n=52) of the participants reported that they assist or assisted the care recipient with money management activities. Userfeel classified 15% (n=17) of participants as “regular users,” 77% (n=85) as “power users” (frequent usability testers who are proficient with various online tools), and 7% (n=8) as “web experts” with a deeper understanding of web design.

### Medicaid Explainer Videos

Most participants found the animated videos helpful in explaining Medicaid concepts and eligibility rules. The mean helpfulness ratings for the videos ranged from 5.9 to 6.4 (out of 7). Participants commented that the videos provided a useful primer on Medicaid but were confused about some of the details, such as whether a spouse’s income is counted toward Medicaid eligibility limits, program differences between states, and rules guiding Medicaid spend down. Others recommended using more images of people:

*You can probably jazz that up a little bit more with some real people in there…and maybe some clickable buttons where you can find more information ‘cuz I still had some questions as I went.*

[45-year-old male]

### Intake Wizard and Medicaid Eligibility Determination

Table 2 displays mean ratings of various website features. Most found it fairly easy to enter the applicant’s financial information requested by the fields (mean 5.9 out of 7, SD 1.34), but based on verbal comments (Multimedia Appendix 3), participants were challenged to enter accurate $ amounts. Some stated that they did not have the care recipient’s detailed financial information on hand or did not have access to their accounts at all. However, most strongly agreed that they understood why they were asked to provide this information (mean 4.9 out of 5, SD 0.43). The average length of time it took participants to complete the intake wizard was 6 (SD 3.03) minutes, although many verbalized that they entered approximations or made up the amounts in the interest of privacy.

**Table 2.** User ratings on web application features (N=109).

Features	Mean (SD)	Rating scale
Explainer videos		
Medicaid overview video	5.9 (1.08)	1=Extremely unhelpful to 7=Extremely helpful
Medicare versus Medicaid video	6.40 (0.77)	1=Extremely unhelpful to 7=Extremely helpful
Income eligibility video	6.18 (1.24)	1=Extremely unhelpful to 7=Extremely helpful
Asset spend down video	6.09 (1.11)	1=Extremely unhelpful to 7=Extremely helpful
Care cost calculator		
Cost estimator rating	6.32 (1.19)	1=Extremely unhelpful to 7=Extremely helpful
Financial data wizard		
Easy to enter financial information	5.86 (1.34)	1=Extremely difficult to 7=Extremely easy
Average website attribute ratings		
Likely to return in the future	4.32 (1.11)	1=Strongly disagree to 5=Strongly agree
Clean and simple presentation	4.65 (0.59)	1=Strongly disagree to 5=Strongly agree
Attractive	4.04 (0.93)	1=Strongly disagree to 5=Strongly agree
Learned about local care costs	4.43 (0.93)	1=Strongly disagree to 5=Strongly agree
Trust website privacy	4.15 (0.94)	1=Strongly disagree to 5=Strongly agree
Information is trustworthy	4.32 (0.79)	1=Strongly disagree to 5=Strongly agree
Information is credible	4.34 (0.79)	1=Strongly disagree to 5=Strongly agree
Videos helped simplify complex information	4.49 (0.80)	1=Strongly disagree to 5=Strongly agree
Helpful for caregivers	4.85 (0.36)	1=Strongly disagree to 5=Strongly agree
Medicaid eligibility rules are clearer	4.53 (0.63)	1=Strongly disagree to 5=Strongly agree
Did not learn anything useful	1.17 (0.55)	1=Strongly disagree to 5=Strongly agree
Understood why I was asked to enter financial information	4.85 (0.43)	1=Strongly disagree to 5=Strongly agree
Experienced technology issues	1.72 (1.34)	1=Strongly disagree to 5=Strongly agree

On the Dashboard (Figure 4), participants were asked whether the information displayed indicated that the care recipient is eligible for Medicaid based on having income below the state limit (55/109, 51.4% said yes) and assets below the limit (42/109, 39.3% said yes). Several participants commented that they appreciated the list of next steps for LTSS planning and how long these steps would take to complete, such as gathering financial statements for the application. However, many described the Dashboard as being too cluttered and “overwhelming,” suggesting a need to simplify the presentation using graphics or reduce the information.

On the Assets page (Figure 5), participants were asked to enter the “spend down” amount displayed or enter US $0 if the care recipient had no spend down obligation because they were currently eligible. Of the total, 89 participants (81.7%) entered the correct number displayed. Of those who failed the task, 2 (1.8% of total) experienced an error message and could not reach the Assets page, 1 (0.9% of total) participant never completed the intake wizard, so the spend down amount could not be calculated, and 11 (10%) could not locate the number displayed.

### Care Cost Calculator

Participants completed 2 tasks on the care cost calculator page (Figure 6). First, they were asked to calculate the average monthly cost of 20 hours per week of home health care and 3 days per week of adult day care in Minneapolis-Saint Paul, Minnesota, and then enter the out-of-pocket cost (US $) that was displayed in the window if the care recipient is enrolled in Medicaid. The amount displayed varied across participants because it is based on the care recipient’s monthly income. Only 59 participants (54.1%) successfully adjusted the settings and entered the number displayed. Reasons for task failure include not selecting the correct state or MSA (n=36), entering the cost displayed for a nursing home instead of in-home care (n=4), entering the wrong hours or days of care (n=5), or some other reason (n=5).

Using the same settings, participants were asked to enter the average cost displayed for in-home care if the care recipient was NOT enrolled in Medicare (ie, private pay). Successful task completion was similar at 56% (n=61). Errors were largely due to not selecting the correct state or MSA (n=30). In cases where the out-of-pocket care costs for private pay matched the out-of-pocket costs for Medicaid-enrolled, the care recipient’s income exceeded the Medicaid eligibility threshold. Participants verbalized that the slider bar was hard to adjust to an exact number because the selection range was large: 0 to 168 hours. Others commented that they did not understand why the cost of home care was not lower if the care recipient was Medicaid-enrolled (vs private pay).

Despite the challenges participants encountered, most rated the care cost calculator as being very helpful (mean 6.3 out of 7, SD 1.19) and strongly agreed that they learned how much long-term care costs in their area (mean 4.4 out of 5, SD 0.93). Many remarked that they were surprised about the high cost of care.

### Improvement in Medicaid Knowledge

Of the total, 93 participants (85%) completed both the Medicaid knowledge pretest and posttest within the 40-minute session. Many participants reasoned through the questions by drawing on their existing knowledge. Those with more experience with Medicaid made comments such as:

*I think depending on the state, you are allowed to own a home/there is five-year look back [period] for Medicaid.*

[60-year-old female]

Using the website and watching 4 of the explainer videos significantly improved participants’ knowledge of Medicaid and Medicaid waiver programs and eligibility criteria (*t*_92_=12.9, *P*<.001). Mean performance on the pretest was 42.4% correct (SD 0.19%), and the mean performance on the posttest was 68.4% (SD 0.15), representing a 61.2% average improvement. To assess the effects of previous Medicaid experience on the change in knowledge score, we assessed differences between participants who care or cared for a Medicaid-enrolled person (n=28) versus participants who care or cared for a nonenrolled person or who were unsure if the person was enrolled in Medicaid (n=56). There were no significant differences between these groups (*t*_68_=0.05; *P*=.96), indicating that watching the explainer videos and interacting with the website yielded equal learning gains for those with and without previous experience with Medicaid. However, it is unknown whether providing care to a person enrolled in Medicaid is an adequate proxy for previous knowledge.

### User Satisfaction

On a scale of 1 (strongly disagree) to 5 (strongly agree), website attractiveness had a mean rating of 4.0 (SD 0.93). It received a higher average rating for having a “clean and simple presentation” (mean 4.7, SD 0.59). One participant stated:

*It’s a lot of information to process but I think it does a good job of guiding you.*

[50-year-old male]

Several participants commented that they wanted to see photographs of diverse older adults to add visual interest to the text-heavy interfaces. The mean rating for trustworthiness of the information was 4.3 (SD 0.79), credibility was 4.3 (SD 0.79), and mean agreement with the statement “I trust the website to keep my responses private” was 4.2 (SD 0.94). In total, 17 participants commented that they were concerned about privacy and were uncertain about how the website intends to use and share the data users enter. A 23-year-old male participant suggested that a privacy disclaimer and a detailed “terms of service” are needed.

Most participants strongly agreed that the website gave them a better understanding of Medicaid eligibility rules (mean 4.5, SD 0.63) and that it would be helpful for caregivers to learn whether a person may be eligible for LTSS benefits through Medicaid (mean 4.9, SD 0.36). Many agreed they would be likely to return sometime in the future (mean 4.3, SD 1.11). Scores were lower among those who shared that the person they cared for is deceased, although some said they would return to explore LTSS options for themselves. Furthermore, 3 participants commented that they wished the website had been available when they were doing care planning for an older relative. Some participants expressed lingering confusion about concepts such as Medicaid spend-down and what assets count toward eligibility limits; however, they recognized that the website covers a challenging topic and providing too many details would be overwhelming. According to one participant,

*...applying for any kind of government help can be daunting and a little scary, so this was a low-pressure way to determine eligibility.*

[66-year-old female]

On average, the total time to complete the user experience testing session was 33 minutes and 43 seconds (SD 5 min 37 s). Participants classified as power users and web experts were more likely to finish all tasks. The average score on the SUS was 88.3 out of 100 (SD 13.1), which is considered very high [33]. Using a slider, participants rated how likely they would be to recommend the website to a friend or colleague. The score was 89.4 out of 100 (SD 18.8), with some stating that they plan to return when they need to apply for Medicaid for themselves or others.

## Discussion

### Principal Results

Based on a gap analysis and resource needs assessment with ADRD caregivers and aging services providers, we designed a web application that reduces information overload by personalizing the Medicaid eligibility and application planning process to the care recipient’s financial circumstances and care preferences. The website received high usability and user satisfaction ratings in the validation and usability test, as well as improved caregivers’ knowledge of Medicaid eligibility rules. Participants commented that the intake wizard was simple to use, yet they recognized the importance of entering accurate amounts to get a precise determination of Medicaid eligibility. If the caregiver is not a joint account holder, accessing the care recipient’s accounts requires them to be legally appointed as a trustee or attorney-in-fact. Another challenge is that accurate data entry requires a relative understanding of complex financial terms and concepts, such as how the cash value of a life insurance policy is different from the death benefit, and that an annuity can be classified as an income source and an asset. While the current web application provides definitions of selected terms when the user hovers over the text, more education on these concepts is needed. A future design could integrate a conversational artificial intelligence–powered agent that helps promote financial literacy, but that is trained not to provide legal or financial advice outside the scope of its service.

We found a statistically significant increase in Medicaid knowledge scores from baseline, suggesting that short, animated videos improve understanding of basic Medicaid concepts. Research indicates that short videos are an effective teaching tool and promote retention [34,35]. Participants verbalized that the videos clarified general Medicaid concepts but that some eligibility rules and exceptions needed elaboration, such as what spousal assets and income sources are counted toward a married applicant’s eligibility. Future research should assess the effects of other presentation formats on learning outcomes, such as infographics and materials that can be printed, translated into multiple languages, and include hyperlinks to detailed external sources.

Findings from this validation and usability study suggest that caregivers are comfortable entering financial information into the website. This is consistent with research showing that individuals may disclose personal information if the web service offers financial benefits or convenience [36] and also supports the “privacy paradox” whereby individuals’ preferences for greater privacy contradict their actual disclosure tendencies [37]. However, some participants expressed concerns about the confidentiality of the information they entered. The development team programmed an account registration feature that was suspended for the validation study to avoid collecting personal identifiers from research participants. Along with providing detailed terms of service, reinstating the login feature is important for securing user data, saving progress, and signaling trustworthiness.

Despite the intake wizard’s simple navigation, participants still encountered errors in calculating eligibility and expressed confusion about asset and income limits. They also verbalized confusion about the amount of income the care recipient must contribute to their care costs once enrolled in Medicaid. These findings underscore the complexity of Medicaid program rules and the confusing LTSS landscape. This complexity has led to a robust market for certified Medicaid planners and elder law or estate planning attorneys, but the cost of these services is greater than most middle- and low-income families can afford [16]. Our web application was designed as a solution to this resource gap.

### Future Development

While the current website provides proof of concept that a digital platform can help informal caregivers learn about and access Medicaid benefits for the care recipient, future development could further personalize the experience and perhaps facilitate the submission of the Medicaid application to the correct state-level authority. The text could be customized to display the Medicaid program name used in the care recipient’s state (eg, “MediCal” for California, “TennCare” for Tennessee) and provide contact information for local care providers that accept Medicaid. For improved accuracy, a more sophisticated eligibility determination algorithm is needed that is automatically updated when state Medicaid policies change and that incorporates additional eligibility exceptions for certain asset types. Many households have complex financial situations, such as assets held in trusts and property located in other states. Determining eligibility for these cases would require the user to enter additional information, adding complexity to the experience. Perhaps a future web application could refer users with complex situations to a local certified Medicaid planner. A future iteration could also integrate a care needs assessment using another intake wizard that collects information on the care recipient’s functional abilities, as these also factor into the eligibility determination.

A recent systematic review found that informal caregivers prefer using a smartphone to access digital tools [38]. In the future, this web application can be redesigned as a smartphone application to improve accessibility. Despite the website’s limitations, many participants, particularly those aged 50 years and older, expressed that they wished the application had been available when they provided care to an older relative. Others shared that they want to return to the website when they are ready to plan for their own care needs. This feedback suggests that there is a strong interest in personalized digital solutions to simplify Medicaid eligibility determinations and learn about regional LTSS costs.

### Limitations

Usability sessions were asynchronous, limiting our ability to ask follow-up questions. Of the total, 2 participants did not complete the intake wizard and encountered error messages that prevented them from completing some of the tasks. This error could be mitigated by limiting navigation options—that is, access to the Dashboard, Assets, and Income pages—before completing the financial intake wizard. Furthermore, 5 participants did not watch all the required videos due to slow loading speeds. This may have affected their satisfaction ratings and scores on the Medicaid knowledge posttest. All multiple-choice and true or false questions were pretested with a separate online panel (n=30), and items were counterbalanced with respect to difficulty and format in the validation study. However, Userfeel does not allow randomization in question and task ordering, which could produce order effects and impact task performance.

Participant confidentiality and an explanation for how data would be used and secured by the researchers were described in the participant consent presented before the usability test. To maintain participant confidentiality, web developers removed the “Create an Account” feature, which would have asked for the participant’s name, email, and a password creation. This account creation page described that no data will be shared with third parties. The absence of a secure login page may have negatively impacted participants’ perceptions of how their information would be secured and shared.

Many participants expressed that they approximated or “made up” asset, income, and monthly spending amounts, affecting the Medicaid eligibility calculation. While accurate entries were not essential for usability testing, accuracy could be enhanced by requiring users to create a login to save their progress, allowing them to return to the website after gathering the applicant’s recent financial statements. The ability to return to the site later without having to start over would also reduce time pressure.

Website validation tests were performed entirely in English by experienced users, limiting the generalizability of the findings. Future validation studies should include inexperienced web users, older caregivers (older than 70 years), and participants with differing levels of experience applying for government programs for themselves or others. A delayed follow-up assessment would also be beneficial to assess knowledge retention of Medicaid concepts.

Although a previous research phase involved usability testing with Spanish-speaking caregivers, further assessment is needed to test the website in other languages. Finally, there were very few spousal caregivers in the sample. Future usability tests should be conducted with spousal caregivers who are typically older than the current participants.

### Comparison With Previous Work

Every state’s Medicaid office has a website with information on program eligibility and application instructions. The Eldercare Locator, a helpline funded by the Administration on Aging, provides guidance on regional aging services providers and resources, as well as a live chat, but does not offer personalized Medicaid eligibility information [39]. AARP’s website offers guidance on getting started with long-term care planning and educates members on Medicare versus Medicaid coverage but does not provide a benefits calculator. The most comprehensive and up-to-date resource on Medicaid LTSS programs was developed by the American Council on Aging [16]. It provides detailed data tables on each state’s income and asset eligibility criteria for married and single applicants, information on Medicaid HCBS waivers by state, rules regarding asset spend down, spousal asset protections, Medicaid estate recovery, nursing home costs, and legal strategies to shield assets. The website also offers a simple eligibility test, but it does not provide an eligibility determination. Rather, users are asked to enter their names and emails to be connected with a Medicaid planner who may charge a service fee. We presented this website to interview participants in the initial resource needs and gap analysis. While caregivers found the site incredibly comprehensive, they described being overwhelmed by the details and the text-heavy interface.

To minimize information overload, we designed our website to limit the amount of information on each page and personalized the eligibility information based on the care recipient’s state and financial circumstances. The videos used simple animations to depict important concepts that affect planning decisions, like spousal asset preservation and Medicaid spend-down strategies. These videos were intended to make Medicaid more approachable for caregivers experiencing high levels of cognitive load and emotional burden. We find that videos are an effective way to improve Medicaid program knowledge, although some content was omitted or simplified. In the future, caregivers would benefit from a digital tool with customized educational videos that is powered to flag financial circumstances that warrant professional advice from an attorney or certified Medicaid planner.

### Conclusions

Medicaid programs provide a critical safety net for millions of older adults and their family caregivers who cannot afford to pay for LTSS out-of-pocket [40]. The application process can be daunting, especially for informal family caregivers with little previous knowledge of Medicaid programs [14,15,19]. Navigating care options and determining how to finance LTSS is very challenging for older adults and their informal caregivers who may be engaging in the process while managing the care recipient’s escalating needs. We designed a website that illuminates the out-of-pocket cost of HCBS and institutional LTSS, educates users on Medicaid programs to help offset these costs, and simplifies eligibility determinations. The encouraging results from the website validation study suggest that there is a clear need for a digital solution to support families in the LTSS and Medicaid planning, but that additional features are needed to educate caregivers about state-specific Medicaid rules, program options, and to streamline the application submission process.

## Supplementary material

10.2196/77441 Multimedia Appendix 1 The first images display photographs from the researcher and developer synchronous problem definition session. The photographs depict a white board with text and sticky notes denoting user needs and possible solutions. Next is a diagram of the initial user journey map created for the first iteration of the application. The last set of images presents 4 example caregiver user personas.

10.2196/77441 Multimedia Appendix 2 Screenshot from a Userfeel testing session. The task window appears in the upper right corner of the participant’s screen. The participant is on the landing page answering a Medicaid knowledge pretest question.

10.2196/77441 Multimedia Appendix 3 Verbal feedback and participant quotes.

## Abbreviations

ADRD: Alzheimer disease and related dementias
HCBS: home and community-based services
LTSS: long-term services and supports
MSA: Metropolitan Statistical Area
SUS: System Usability Scale
