# Supplementary material for: Medicaid Education and Eligibility Planning for Caregivers: Website Usability and Validation Study
Source: JMIR Aging. 2025 Aug 27;8:e77441. doi: 10.2196/77441 (PMC12386547; doi:10.2196/77441)

**Multimedia Appendix A. Initial website development activities**

**Figure A. Photographs from the researcher and developer synchronous problem definition session**

**
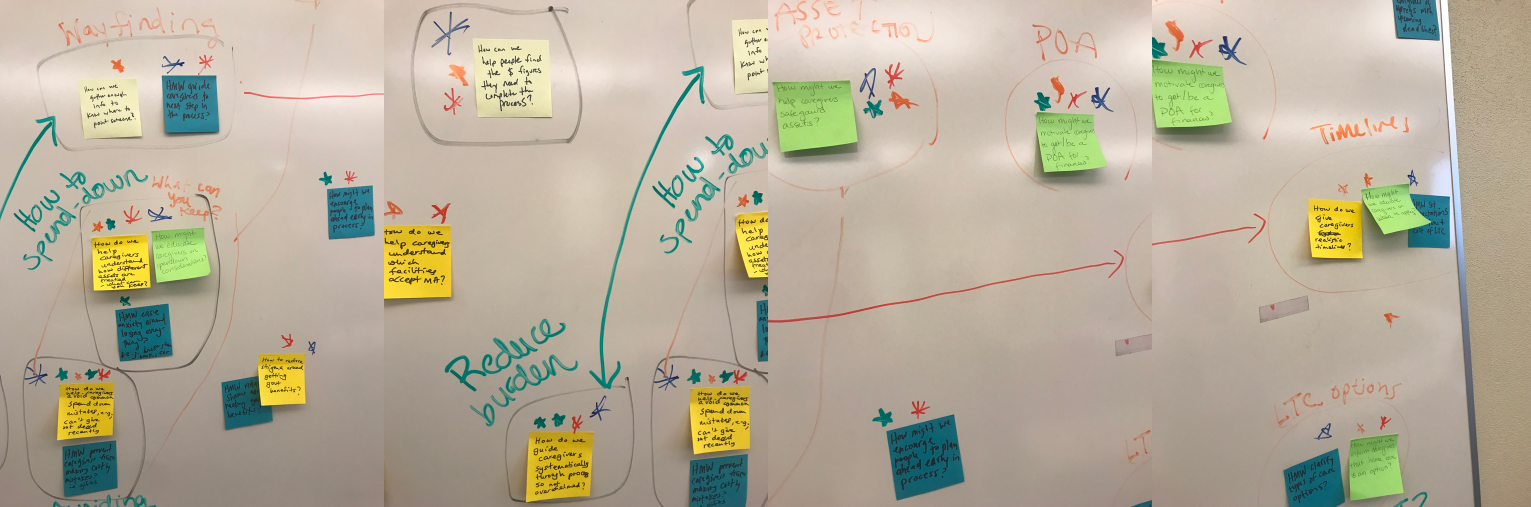
**

**
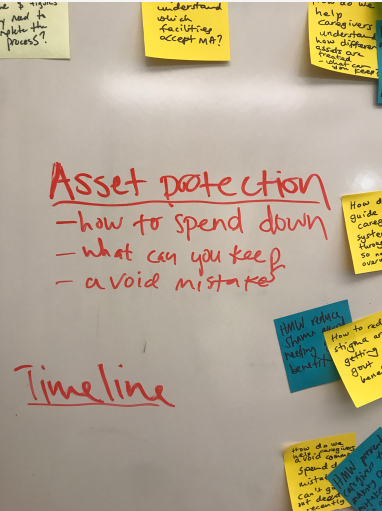

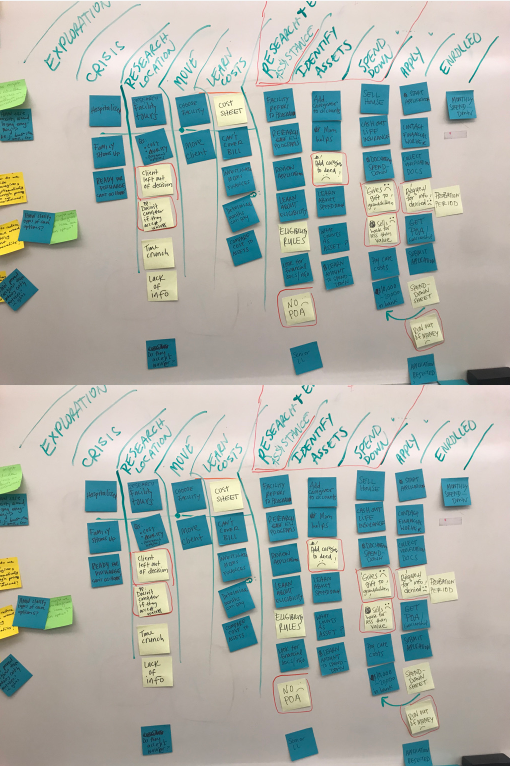
**

**Figure B. Diagram of the initial user journey map used for first iteration of the application**


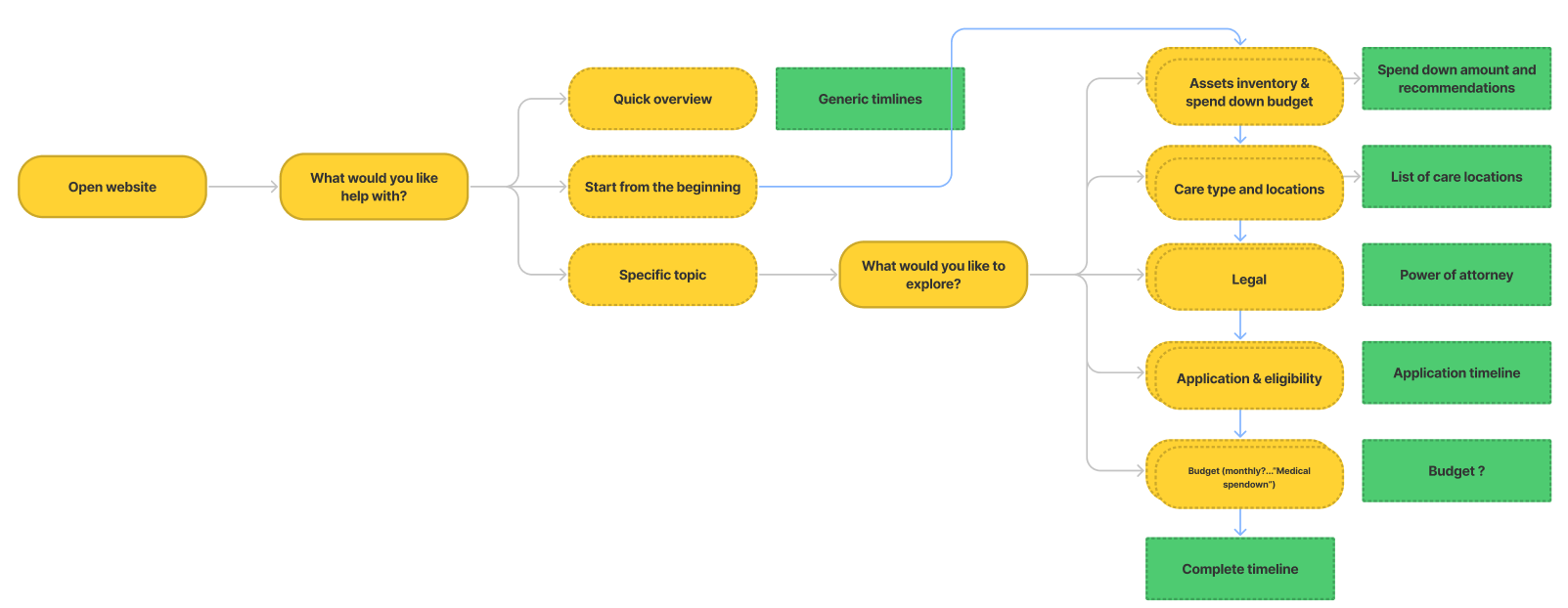


**Figure C. Example caregiver user personas**


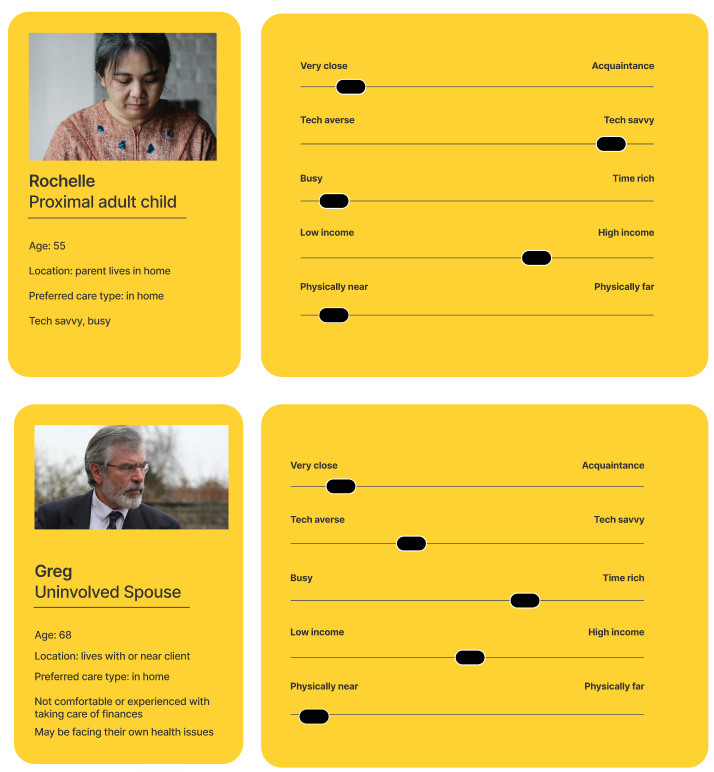

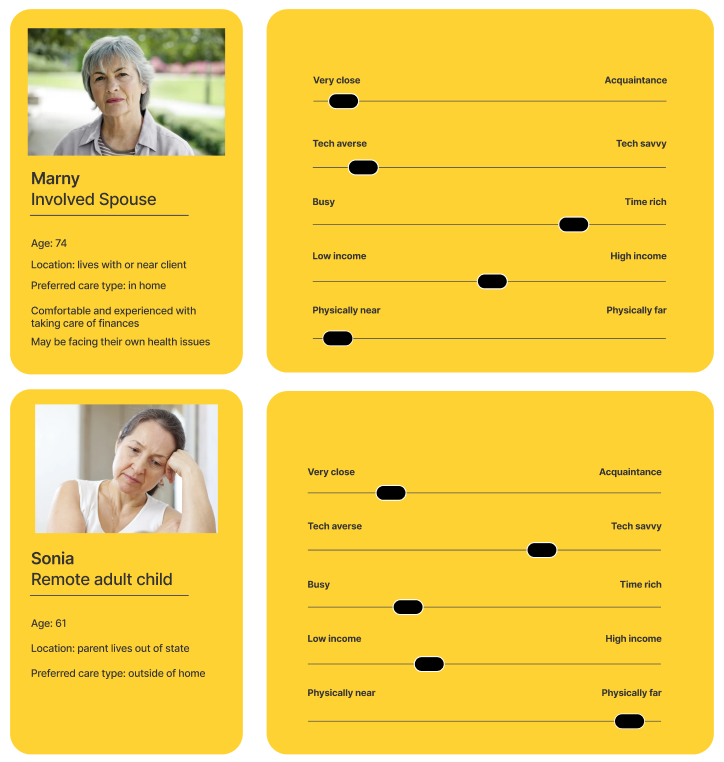

Supplement: Multimedia Appendix 1 [file aging-v8-e77441-s001.docx]
