# Supplementary material for: Medicaid Education and Eligibility Planning for Caregivers: Website Usability and Validation Study
Source: JMIR Aging. 2025 Aug 27;8:e77441. doi: 10.2196/77441 (PMC12386547; doi:10.2196/77441)
Supplement: Multimedia Appendix 2 [file aging-v8-e77441-s002.docx]

**Multimedia Appendix 2. Screenshot from a Userfeel testing session**


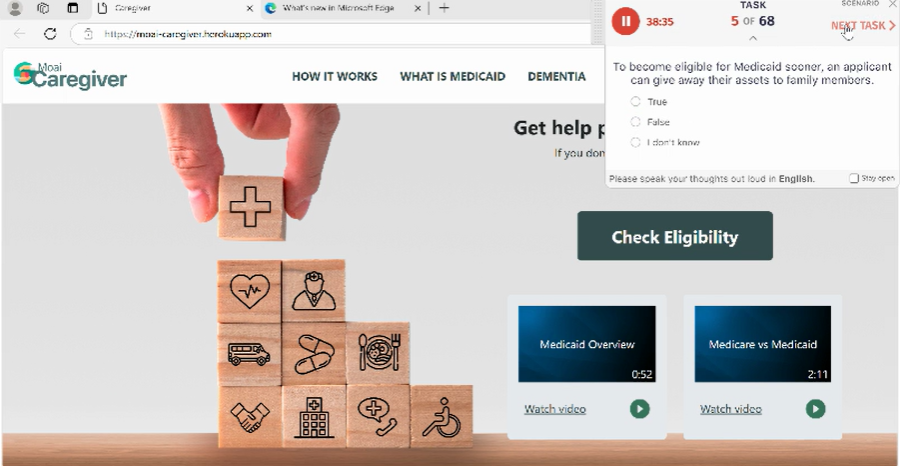


Note: Task window appears in the upper right corner of the participant’s screen. The participant is on the landing page answering a Medicaid knowledge pretest question.
