# Supplementary material for: Medicaid Education and Eligibility Planning for Caregivers: Website Usability and Validation Study
Source: JMIR Aging. 2025 Aug 27;8:e77441. doi: 10.2196/77441 (PMC12386547; doi:10.2196/77441)
Supplement: Multimedia Appendix 3 [file aging-v8-e77441-s003.docx]

**Multimedia Appendix 3. Qualitative feedback on website features with participant quotes**

| **Feature** | **Summary of participant feedback** | **Example quotes: Positive** | **Example quotes: Negative** | **Example quotes: Improvement suggestions** |
| --- | --- | --- | --- | --- |
| **Medicaid explainer videos** | 1. Videos helped participants understand general Medicaid concepts  2. Still some confusion, more details could be helpful  3. Suggestions included ending videos with links to check state services or find more detailed information, adding subtitles, and adding the option to reduce video speed | Medicare vs. Medicaid video: "I thought the Medicare versus Medicaid video was a good succinct discussion about the differences between the two services...It was supposed to paint in broad brushstrokes the differences and it did point out that Medicare does not pay for long-term care; Medicaid is limits tested, in terms of assets. It did talk about the different populations that the two are targeting, so I would say that was extremely helpful" (MP_S6, F, 61). | Asset spend down video: "I would say this video was helpful, but not completely helpful. I found it still a little bit confusing about how, on the one hand, the spouse is allowed to keep their own assets basically, in essence, but their assets are counted towards eligibility. So it seems to me like that's almost contradictory and that if you're allowed, I don't understand how those two statements mesh. I really could've used more of an explanation" (MP_S6, F, 61). | Medicare vs. Medicaid video: "I would like to say that since the video ends on that note to check for, you know, state-specific services...that it would be nice to have a link to those different states and what they provide, because if it's a old person or someone not very familiar with how to get that information they might not know how to get it or just not be willing to go searching for it." (MP_S5, F, 27). |
|  |  | Income eligibility video: "The video was understandable and it actually gave me some good facts that I did not know actually. I think a little bit more explanation in why they only get to keep $125 of their income, but otherwise I think it was helpful" (MV2_S5, F, 23). | Medicaid overview video: "It was too basic as well. There were, there are more parts and things that needs to be clarified" (MP_S8, M, 41). | General feedback on videos: "We can include subtitles that would really help, because sometimes people might have some disability that would prevent them from hearing or something, so you know we should really have that captions button underneath and if we could reduce the speed of the video because it was too fast... I did understand everything, but still it was faster than the normal videos that we see of similar kind" (MF_S7, F, 35). |
|  |  | Asset spend down video: "I'm still a little confused, but it was definitely very helpful" (MV3_S20, F, 56). |  | Spend down video: "It was helpful, it's just there's more that I feel like I don't know about it. It was helpful seeing the burial plots and stuff like that. I wish there was like, 'for further questions go here.' Something to give me more" (MV2_S59, F, 34). |
| **Financial intake wizard** | 1. Participants generally found the text readable, and the asset and income types clear  2. Main challenge was knowing the financial details of the care recipient/applicant (i.e., exact amounts of their bills and money in accounts)  3. Multiple suggestions to provide a list of information needed ahead of time so users can gather documents before starting the intake wizard | "The website consistently has done an amazing job at keeping things clear, large font, which is also important when reviewing this type of information with the elderly. It kept the calculations or the calculator portion very simple and straightforward, so I'm going to again rate it extremely easy [to use]" (MV2_S84, F, 49). | "You know what, a little confusing in terms of insurance versus home expenses, but honestly, a lot of that is just having to read through it a second time and then it was fine" (MF_S11, M, 62). | "I kind of wish they'd given me a list of what I was gonna need like to gather documents, like it has on the side here, now it says 'Next Steps.' Well, I needed that earlier to determine what I needed because I was sitting here trying to figure everything up in my head because I didn't have her bills in front of me, and her insurance premium, and her all that and the other. So it wasn't hard to input it, it was just hard to figure it out off the top of my head" (MV2_S85, F, 56). |
|  |  | "It was pretty easy to enter that information, the financial information, as long as you have that available" (MV3_S6, M, 59). | "It was easy to answer it but it was hard for me to think...If a person doesn't know a person that well, or, but I'm assuming you'll be next to this person or like knowing this person way better than someone that's just a friend that took care of him" (MV2_S58, M, 34). | "Maybe you should have something at the beginning of this that says, 'Here's the kind of information you're going to have to get together before you start this process'" (MV3_S2, M, 63). |
|  |  |  |  |  |
| **Dashboard** | 1. Multiple participants expressed there was too much information on one page  2. Participants appreciated that the Dashboard showed them the next steps in the planning process | "'Learn how to still qualify.' This is something that I would be interested in for myself in the future. I would really like to have a website like this" (MP_S6, F, 61). | "I got so confused, it's so much stuff I want to read" (MP_S3, F, 34). | "It's good, it's informative, but the arrangement is a little... too much on one screen, it should be different screens rather than just put it on one. It can be overwhelming the way they have it arranged" (MF_S19, F, 53). |
|  |  | "State monthly income is below limit, it's right there. It's very easy to see, it's highlighted. It's right at the top of the page" (MV2_S76, F, 60). | "It got a little confusing, like I said they have too much stuff on one page" (MF_S14, F, 40). |  |
|  |  | "That's pretty helpful and has a plan for your next steps" (MV3_S2, M, 63). |  |  |
| **Care cost calculator** | 1. Calculator was described as helpful  2. Some participants were confused about what the numbers actually meant in terms of what Medicaid pays and what costs the person seeking care would still be responsible for  3. One participant thought it would be better to enter the exact number of hours of home care desired instead of using slider bar | "I think it was extremely helpful, actually" (MF_S18, M, 30). | "The only thing I guess I got confused on was...the private-pay and Medicaid enrolled, I was a little confused in the beginning as to whether this was what Medicaid paid or what he paid, but then after the second question I understood what it was" (MF_S2, F, 53). | "I think it would be nicer if it did have the ability to put the exact number in [instead of using the slider bar]" (MV2_S83, M, 41). |
|  |  | "I loved the calculator, it's always helpful, I like to see solid numbers" (MV2_S57, F, 63). | "It's a simple tool okay, but not quite fully understanding if it's Medicaid enrolled. Does that mean Medicaid pays that [amount] and the person pays zero? ...if Medicaid is going to cover that cost and it's going to be less expensive overall, then ok I understand that...It is a little confusing" (MV2_S78, M, 45). |  |
|  |  | "Wow, that just blows my mind [Medicaid enrolled is $200]! That's cool, and wow it [private pay] would cost over $4000" (MV3_S15, F, 62) |  |  |
| **Visuals and overall design** | 1. Participants did not find the website to be exceptionally visually appealing, but commented that it is more important to prioritize functionality over aesthetics  2. Participants found the visual aspects to be practical  3. One person recommended using more underlining and bold fonts to emphasize important information; others wanted more pictures | "It's not the most aesthetically pleasing thing in the world, but, for what it is, you know, it gives you a very plain, clear, concise look at the information that you need, but that's all it really needs to be" (MV2_S54, M, 22). | "[The website] is a little dry, there could be a little color" (MV2_S1, M, 54) | "I mostly find it to be attractive, but I do think that adding a little bit more of diversity would help, you know, digest information a little easier. Even just things like underlining, especially when it comes to headings or, maybe using bold for some of the terms to make them stand out, things like that" (MF_S15, F, 38). |
|  |  | "I don't need a website to be attractive really, just need it to work...It's got pretty clean lines...it's no more cluttered than it needs to be" (MV3_S2, M, 63). | "[The website] has too many numbers on the screen; [makes it] seems less attractive " (MV2_S61, M, 35) |  |
|  |  |  |  |  |
